# Genomic variants in Fas-mediated apoptosis pathway predict a poor response to Platinum-based Chemotherapy for Chinese Gastric Cancer Patients

**DOI:** 10.7150/jca.48120

**Published:** 2021-01-01

**Authors:** Tingting Zhao, Wei Li, Jinfei chen, Weisong Qin

**Affiliations:** 1 National Clinical Research Center of Kidney Diseases, Jinling Hospital, Nanjing University School of Medicine, Nanjing, 21000, China.; 2 Department of Gynecology, Zhenjiang Maternity and Childcare Hospital, Zhenjiang, 212000, China.; 3 Cancer Center, Taikang Xianlin Drum Tower Hospital, Nanjing University School of Medicine, Nanjing, 21000, China.

**Keywords:** Fas-mediated apoptosis, gastric cancer (GC), genetic variants, chemotherapy

## Abstract

Platinum-based adjuvant chemotherapy is very common for gastric cancer (GC) patients, but the chemotherapy sensitivity is very heterogeneous. The genomic variants and the gene-gene interactions involved in Fas-mediated apoptosis pathway including Fas (FAS 1377 G > A and 670 A > G), FasL (FASL 844 C > T) and caspase-8 (CASP8 -652 6N ins > del or I > D), may paly vital roles in the response to platinum-based treatment. In our investigation, 662 stage II-III postoperative GC patients were enrolled between 1998 and 2006. 261 patients accepted platinum-based regimens and the remaining 401 were not. The log rank tests, Kaplan Meier plots, Pearson chi-square tests, Student t-tests and Cox regression analyses were performed. For the chemotherapy cohort, FAS 1377 G > A or FAS 670 A > G variants alone was related with inferior survival, and a greater than additive effect was identified when patients simultaneously carrying FAS 1377 GA and FAS 670 GA genotypes. But the poor response was neutralized when patients simultaneously carrying FASL 844 C > T or CASP8 -652 6N ins > del mutations. Our study suggested that FAS 1377 G > A and FAS 670 A > G variants may serve as potential biomarkers to predict the response to platinum-based adjuvant chemotherapy, and the gene-gene interactions involved in Fas-mediated apoptosis pathway may enhance or neutralize the chemosensitivity.

## Introduction

In spite of the mortality is decreasing in the past few decades, gastric cancer (GC) still represents the second most common cancer-related deaths all over the word, particularly in developing countries 1-3. Surgery is the main treatment for GC patients, but two thirds of GC patients recur after curative resection 4. So, postoperative chemotherapy is globally considered as the standard treatment in clinic 5. The 5-year survival rate for the cancer patients accepted postoperative chemotherapy extends from 49.6% to 55.3% compared with tumor resection alone 6. Up to now, although novel therapeutic strategies have been developed rapidly, platinum-based chemotherapy remains the best choice for GC patients 7. But the clinical therapy benefits are various mainly due to the differences in chemosensitivity. Genetic variants involved in Fas-mediated apoptosis pathway may exert crucial roles in the treatment effect for platinum-based adjuvant chemotherapy 8,9. Thus, identifications of the single-nucleotide polymorphisms (SNPs) and the gene-gene interactions involved in Fas-mediated apoptosis pathway are of great importance to make more precise evaluation of the chemotherapy efficacy and then design personalized therapy.

Apoptosis, also named programmed cell death (PCD), is relate to diverse biological processes, such as the maintenance of tissues homeostasis, the development of cell proliferation, the disorder of immune surveillance, and the elimination of cancer cells 10,11. And the abnormal regulations of apoptosis can lead to numerous human diseases, including cancer 12,13. As we all known, apoptosis mainly occurs through two signaling pathways, including the extrinsic pathway and the intrinsic pathway. Fas/FasL system triggered extrinsic pathway is a main mechanism for the induction of cell and tissue apoptosis 14. Fas (APO-1TNFSF6/CD95) interacts with FasL (CD95L) to initiate the death signal cascade and consequentially actives caspase-8 (CASP8), eventually leading to cell death 15,16. The activations of Fas-induced apoptosis may exert essential functions in the treatment of platinum-based chemotherapy, and the dysregulations of the Fas/FasL signaling pathway might affect the chemosensitivity 17,18. The decreased Fas level might down-regulate of Fas-induced apoptosis, while the elevated FASL level would enhance the ability of cancer cells to fight against immune system by killing FAS sensitive lymphocytes, which might subsequently affect chemotherapeutic response and change the prognosis of patients accepted chemotherapy 19,20.

FAS gene is located on chromosome 10q24.1, which is a cell-surface receptor of tumor necrosis factor (TNF) receptor superfamily 21. FAS is widely expressed in various tissues and interacts with FASL to active apoptotic signaling pathway 22. FASL gene (another TNF receptors), mapped on chromosome 1q23, is mainly expressed in cytotoxic T lymphocytes and natural killer cells 23. CASP8 gene, situated in chromosome 2q33-34, is the key regulator of the FAS/FASL-induced apoptosis in T lymphocytes. And CASP8 may exert critical roles in the defense mechanisms against hyper-proliferation and tumorigenesis 24.

Recently, some novel SNPs in the promoter regions of FASL, FAS and CASP8 have been identified. The FAS 670 A > G (rs1800682 A > G) and 1377 G > A (rs2234767 G > A) variants could disrupt Sp1 (Transcription factor Sp1) and STAT1 (Signal transducer and activator of transcription 1) transcription factor binding sites, which may reduce the level of Fas gene and diminish its promoter activity 25. In regard to FASL, the transition from C to T at position 844 (rs763110 C > T) creates a new binding motif for another transcription factor, CAAT/enhancer-binding protein β, which may lead to obvious reduction of FASL 26. As for CASP8, a six-nucleotide deletion variant of AGTAAG at position 652 (-652 6N ins > del or I > D, rs3834129) in the promoter region may destroy the Sp1 transcriptional activator binding element 27. So, CASP8 -652 6N ins > del genetic polymorphism could decrease CASP8 mRNA expression and diminish the activity of CASP8, which may significantly reduce T lymphocytes apoptosis and obviously improve immune surveillance 28.

Previous investigations demonstrated that those functional genetic variations of Fas/FasL/CASP8 may influence the susceptibility to gastric cancer or affected the sensitivity to platinum-based chemotherapy for some types of cancer, including non-small-cell lung cancer, malignant pleural mesothelioma and so on 29-31. However, the associations between genomic variants involved in Fas-mediated apoptosis pathway, with the response to platinum-based chemotherapy for gastric cancer patients, was still unclear. In addition, whether the interactions between the genes could enhance or neutralize the sensitivity to platinum-based treatment was also confused. So, for the first time, we systematically investigated the individual and synergistic effects of the variants of FASL, FAS and CASP8 on the response to platinum-based adjuvant chemotherapy for postoperative stage II-III GC patients on the basis of large amount of clinical data.

## Methods

### Enrolled patients

The retrospective cohort study was approved by the Institutional Review Committee of Nanjing Medical University (Nanjing, China). Patients enrolled in our investigation must meet the following criteria: a) histopathological and imaging diagnosed as stage II-III GC; b) no radiotherapy or chemotherapy before surgery; c) the clinical data were complete without missing; d) signed an informed consent to use postoperative tissues for clinical researches; e) conducted operation at the Yixing People's Hospital (Yixing, Jiangsu Province, China) from January 1998 to December 2006; f) accepted follow-up after surgery. Above all, a total of 662 stage II-III GC patients were collected, and the patients' general clinical and pathologic characteristics were summarized in Table 1. Within one month after operation, 261 recruited patients accepted platinum-based chemotherapy, but the other 401 had not due to different reasons. Chemotherapy was given when the patients reached the following level: neutrophil counts ≥ 1.5× 10^9^/L, hemoglobin level ≥ 8 g/dl, platelet counts ≥ 100 × 10^9^/L, as well as no symptom of organ toxicities. If necessary, Antiemetics and mannitol diuresis were used. The follow-up period was counted from the operation date to the death or last follow-up time (March 31, 2009, up to 119.0 months), and the median follow-up time was 68.5 months. The TNM staging were assessed on the basis of the TNM classification of the American Joint Committee on Cancer (7th edition). The histopathology of GC was classified into diffuse and intestinal types on the basis of Lauren's Criterion 32. And all of our researches were based on the clinical guidelines of Nanjing Medical University.

### Genotyping

The methods that extract genomic DNA from paraffin sections were similar to previous description 33. The above genes variants were measured by SNaPshot using an ABI fluorescence-based assay allelic identification method or automated sequencing. Supplementary Table S4 listed the primer sequence of each SNP. The polymorphisms were determined using an ABI3130 genetic analyzer and the Genemapper version 4.0 was adopted to analyze genotypes (Applied Biosystems, Forster City, CA) 34. Two people randomly selected 10% of the samples to independently verify the genotype the results were 100% consistent.

### Statistical analysis

When the median survival time (MST) cannot be got, we chose mean survival time instead. Student t-tests or the Pearson chi-square tests was adopted on the basis of the variable type to elucidate the correlations between each and/or combined genotypes with clinicopathologic parameters. Kaplan-Meier plots and log-rank tests were calculated by SPSS 20.0 software (Inc, Chicago, IL, USA). The estimation of the crude/adjusted hazard ratios (HRs) and 95% confidence intervals (95% CIs) were calculated by univariate or multivariate Cox regression analysis. All of the above tests were bilateral and *P* < 0.05 was defined as statistical significance.

## Results

### Clinical features of the study subjects

**Table 1** showed the clinical features of the 662 recruited GC patients in II-III phase. The median age was 61.0 years (range 28 to 83 years). During the total number of 119.0 months follow-up, 335 patients died. 261 patients received platinum-based adjuvant chemotherapy, and the other 401 did not. For the cohort did not accept platinum-based treatment, the overall survival time was closely related to histological type, tumor differentiation, invasion depth, Lymph node metastasis and TNM stage (*P* < 0.05). In particular, the mortality rate of no-chemotherapy patients with diffuse-type (MST, 37 months) was 54.5% notably higher (HR = 1.545, 95% CI = 1.1125-2.122, *P* = 0.006) than that of patients with intestinal type (MST, 74 months). Compared with patients with poor differentiation (MST, 41 months) and mucinous or signet-ring cell (MST, 32 months), patients with well differentiated (MST, 70 months) had 36.9% (HR = 1.369, 95% CI = 1.000-1.875) and 116.5% (HR = 2.165, 95% CI = 1.224-3.829, *P* = 0.015) reduced death risk, respectively. Patients with T3/T4 invasion depth (MST, 48 months) had 64.7% higher death risk (HR = 1.647, 95% CI = 1.002-2.706, *P* = 0.045) than those with T1/T2 invasion depth (MST, 75 months). Patients with N1/N2/N3 lymph node metastasis (MST, 41 months) had 46.5% higher death risk (HR = 1.465, 95% CI = 1.032-2.080, *P* = 0.030) than those with no lymph node metastasis (MST, 67 months). Meanwhile, Patients in TNM stage III (MST, 37 months) had 91.7% higher death risk (HR = 1.917, 95% CI = 1.394-2.637, *P* < 0.001) when compared with those patients in stage II (MST, 75 months). However, no similar higher death risk was identified in the chemotherapy cohort.

### FAS 1377 G > A and FAS 670 A > G variants contributed to a poor response to platinum-based chemotherapy

We applied the Cox regression analyses in various genetic models to assess the associations between the genetic variants involved in the Fas-induced apoptosis pathway with the overall survival among the two cohorts (**Table 2**). For the patients underwent chemotherapy, significantly reduced survival time were observed for FAS 1377 G > A and 670 A > G mutations in codominant and dominant models.

**Figure 1:**
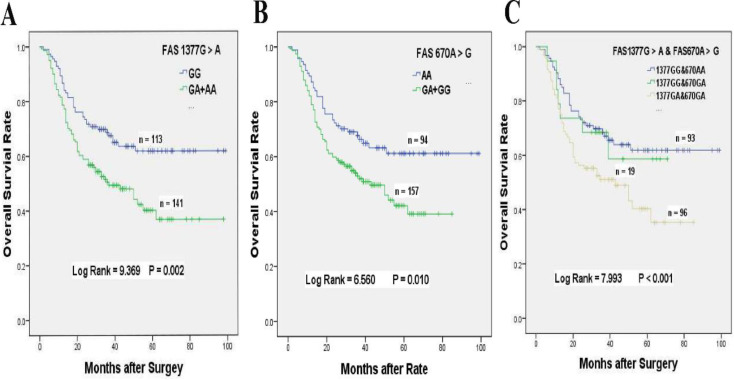
Overall survival of *FAS 1377 G > A* and/or* 670 A > G* polymorphisms for GC patients accepted platinum-based chemotherapy. **(A)** Kaplan-Meier survival curve of *FAS 1377 G > A* in dominant genotypes for overall survival of GC patients received chemotherapy. **(B)** Kaplan-Meier survival curve of *FAS 670A > G* in dominant genotypes for overall survival of GC patients received chemotherapy. **(C)** Kaplan-Meier survival curve of FAS 1377 G > A interaction with 670 A > G in heterozygous genotypes for overall survival of GC patients received chemotherapy.

Our results demonstrated that chemotherapy patients with FAS 1377 GA + AA genotypes had 80.1% significantly higher death risk than those carrying GG genotype (HR = 1.801, 95% CI = 1.225-2.648, *P* = 0.002, **Table 2, Figure 1A**). The MST of 1377 GA + AA genotypes was shortened from 69 to 51 months when compared with GG genotype. Similarly, chemotherapy patients with FAS 670 GA + GG genotypes also exhibited 67.6% obviously higher death risk than those carrying AA genotype (HR = 1.676, 95% CI = 1.120-2.507, *P* = 0.010, **Table 2, Figure 1B**). Compared with AA genotype, the MST of 670 GA + GG genotypes were shortened from 68 to 43 months.

### Prolongation of no chemotherapy patients' survival time with CASP8 -652 6NI > D variant

As for CASP8 -652 6N I > D variants, we observed that, in all genetic models, CASP8 -652 6N I > D mutation obviously protected untreated GC patients from death in all genetic models. No chemotherapy patients with 652 6 DI + DD genotypes exhibited obviously reduced death risk by 31% (HR = 0.690, 95% CI = 0.518-0.921, *P* = 0.010, **Table 2**) compared with those carrying II genotype. The MST in patients carrying DI + DD genotypes was prolonged from 36 to 65 months when compared with those with II genotype. In addition, untreated GC patients with codominant or recessive models also showed remarkably longer survival time.

No significant relationships were identified between other polymorphisms including CASP8 -652 6N I > D and FASL 844 C > T with the survival of GC patients accepted platinum-based treatment in any genetic models. Furthermore, none of the remaining three genes polymorphisms except CASP8 -652 6N I > D were associated with the overall survival for the no chemotherapy patients.

### Stratified analyses of FAS 670 A > G and 1377 G > A variants on the increased susceptibility to chemoresistance

**Figure 2:**
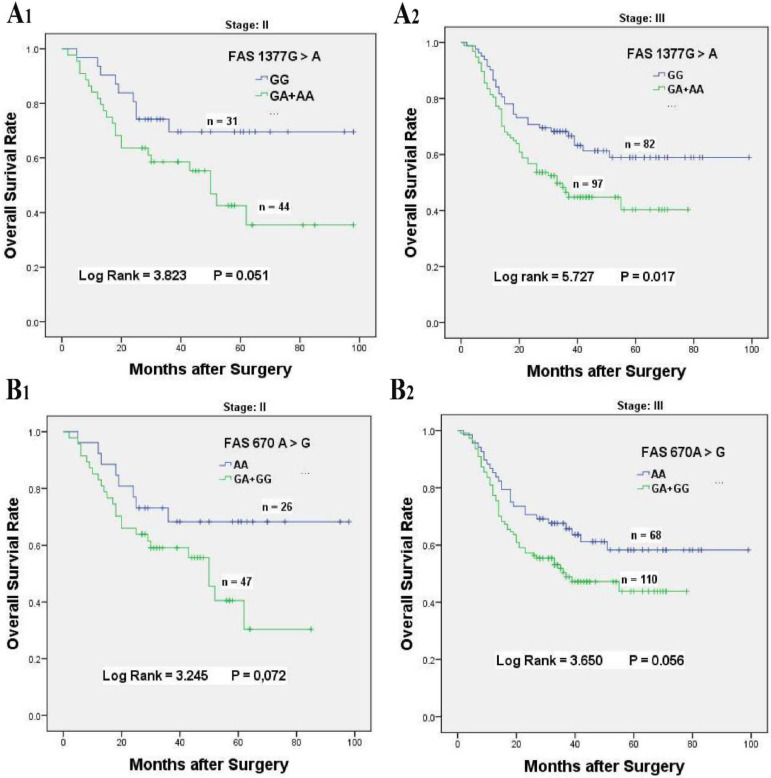
Kaplan-Meier survival curves of *FAS 1377 G > A* and* 670 A > G* for overall survival of stage II-III GC patients accepted platinum-based chemotherapy. **(A)** Overall survival of *FAS 1377 G > A* in dominant genotypes for stage II (A_1_) and stage III (A_2_) chemotherapy patients, respectively. **(B)** Overall survival of *FAS 670 A > G* in dominant genotypes for stage II (B_1_) and stage III (B_2_) chemotherapy patients, respectively.

To better explore the associations between FAS 670 A > G and 1377 G > A variants with the overall survival of patients received chemotherapy, stratified analysis was performed based on the ages, tumor sizes and sites, tumor differentiation, histological types, lymph node metastasis, invasion depth, metastasis distant and TNM stage (**Table 3**). For FAS 1377 G > A variant, compared with the GG genotype, the FAS 1377 GA + AA genotypes were obviously associated with poor survival for chemotherapy patients in age ≤ 60 (HR = 1.981, 95% CI = 1.181-3.323, *P* = 0.008), tumor size > 5 cm (HR = 2.056, 95% CI = 1.134-3.726, *P* = 0.014), tumor arising at Non-cardia (HR = 2.011, 95% CI = 1.220-3.314, *P* = 0.005), tumor of diffuse type (HR = 1.791, 95% CI = 1.118-2.869, *P* = 0.013), poorly differentiated tumor (HR = 1.805, 95% CI = 1.064-3.060, *P* = 0.025), stage T3/T4 invasion depth (HR = 1.851, 95% CI = 1.225-2.795, *P* = 0.003), N1/N2/N3 lymph node metastasis (HR = 1.678, 95% CI = 1.108-2.541, *P* = 0.013), no distant metastasis (HR = 1.801, 95% CI = 1.225-2.646, *P* = 0.002) and TNM stage III (HR = 1.707, 95% CI = 1.092-2.670, *P* = 0.017, **Table 3, Figure 2A_1_ & 2A_2_**).

Similarly, stratification analysis for FAS 670 A > G polymorphism also showed that GA + GG genotypes had distinct relationships with the inferior survival for patients with age ≤ 60 (HR = 1.852, 95% CI = 1.076-3.188, *P* = 0.032), tumor arising at non-cardia (HR = 1.806, 95% CI = 1.067-3.055, *P* = 0.024), tumor of intestinal type (HR = 2.475, 95% CI = 1.192-5.140, *P* = 0.011), well to moderately differentiation (HR = 2.087, 95% CI = 1.092-3.989, P = 0.022), stage T3/T4 invasion depth (HR = 1.715, 95% CI = 1.115-2.637, *P* = 0.012), and N1/N2/N3 lymph node metastasis (HR = 1.537, 95% CI = 1.00-2.372, *P* = 0.048, **Table 3, Figure 2B_1_ & 2B_2_**).

### The gene-gene interactions influenced the overall survival of GC patients accepted platinum-based adjuvant chemotherapy

**Figure 3:**
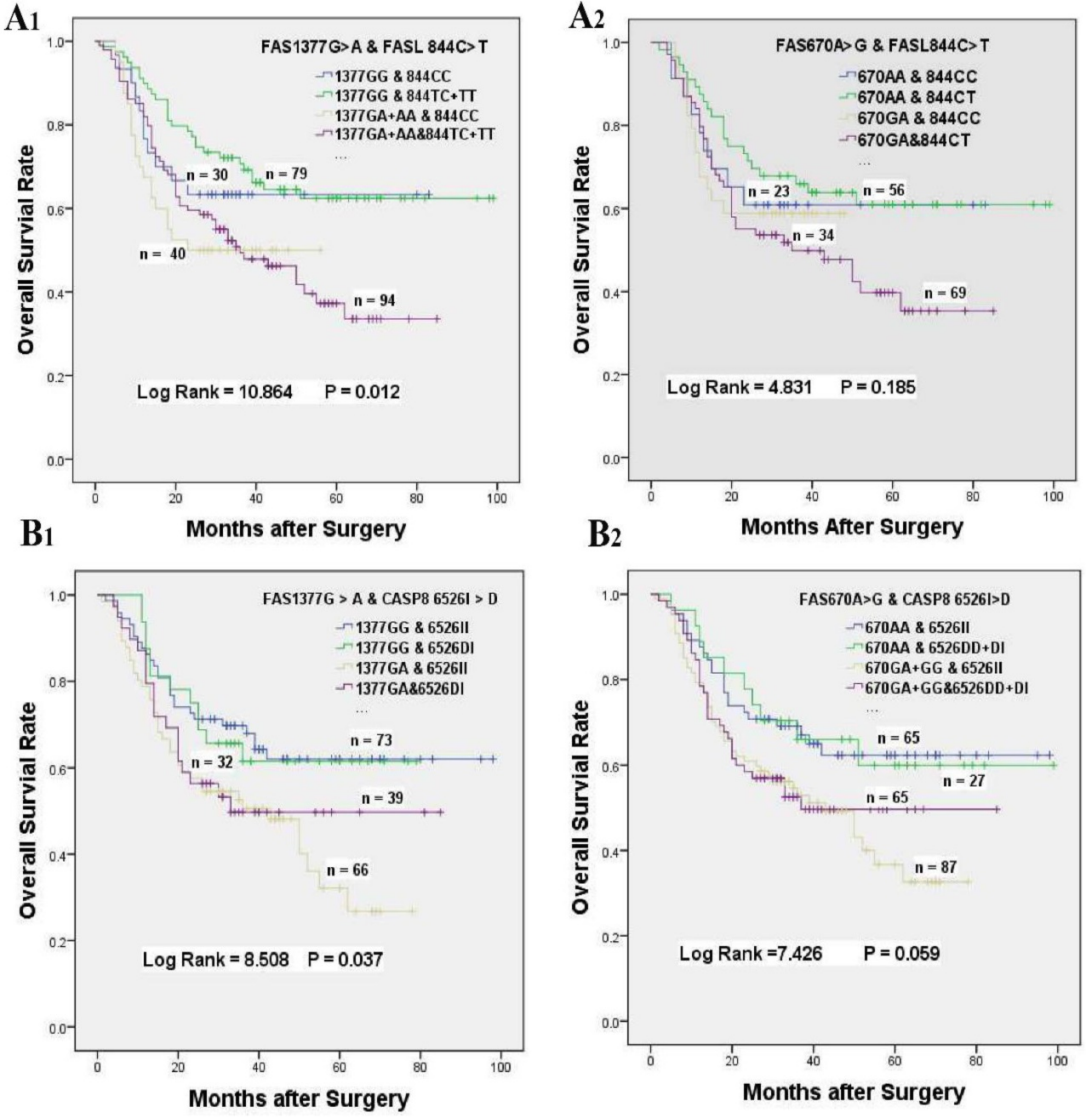
The effects of gene-gene interactions on the overall survival of GC patients accepted platinum-based chemotherapy. **(A)** Kaplan-Meier survival curves of FASL 844 C > T interaction with FAS 1377 G > A (A_1_) or FAS 670 A > G (A_2_) on the survival of GC patients underwent chemotherapy. **(B)** Kaplan-Meier survival curves of CASP8 652 6N I > D interaction with FAS 1377 G > A (B_1_) or FAS 670 A > G (B_2_) on the survival of GC patients underwent chemotherapy.

In consideration of the complex interactions between the above genes, we focused on the effects of the gene-gene interactions on the prognosis of GC patients underwent platinum-based treatment (Supplementary Table S1-S3). In univariate analysis, FAS 670 A > G or 1377 G > A variants alone was related to inferior survival, and a greater than additive effect was identified when patients simultaneously carrying FAS 1377 GA and FAS 670 GA genotypes (HR = 1.830, 95% CI = 1.179-2.840, log-rank *P* < 0.001, **Table 4, Figure 1C**). Whereas, the poor survival of patients carrying FAS 1377 GA + AA or FAS 670 GA genotypes disappeared when GC patients simultaneous with FASL 844 TC + TT or FASL 844 TC genotypes, which were not related to the survival of GC patients accepted treatment in univariate analysis (HR = 1.449, 95% CI = 0.765-2.777, *P* = 0. 012; HR = 1.384, 95% CI = 0.670-2.860, *P* = 0.185, respectively, **Table 4, Figure 3A_1_ & 3A_2_**). Meanwhile, the inferior survival of FAS 1377 GA were maintained when patients simultaneously carrying CASP8 -652 6N II genotype (HR = 1.918, 95% CI = 1.164-3.162), but the shortened survival time vanished when patients also carrying CASP8 -652 6N DI genotype (HR = 1.627, 95% CI = 0.899-2.945, *P* = 0.037, **Table 4, Figure 3B_1_**). The similar results were existed when patients simultaneously carrying FAS 670 GA + GG and CASP8 -652 6N II or CASP8 -652 6N DD + DI genotypes (HR = 1.790, 95% CI = 1.086-2.950 and HR = 1. 630, 95% CI = 0.948-2.801, *P* = 0.059 respectively, **Table 4, Figure 3B_2_**).

## Discussion

Our present research systematically explored the clinical significances of the SNPs related to the Fas-induced apoptosis pathway and the gene-gene interactions on the prognosis of GC patients in II-III stage that underwent platinum-based adjuvant chemotherapy. Our investigations demonstrated that FAS 670 A > G and 1377 G > A mutations contributed to a poor response to platinum-based treatment, and the treatment efficacy was also interfered by gene-gene interactions. So, our investigation provided the useful basics to make more precise evaluation of the chemotherapy efficacy and then further design personalized therapy.

The FAS 670 A > G and 1377 G > A variants could decrease Fas level and diminish Fas promoter activity, thereby down-regulating Fas-mediated apoptosis 21,35. Previous meta-analysis has indicated that FAS 1377 G > A mutations might increase Asian sensitivity to GC, but no significant associations were identified between FAS 670 G > A variant and GC risk 29,36. Besides, the genetic polymorphisms involved in Fas-induced apoptosis pathway might also play significant effects on the prognosis of some types of cancer patients underwent platinum-based chemotherapy 19,20,37, 38. For example, Li et al. indicated that the prognosis (PFS and OS) of FAS 670 A > G polymorphism GG genotype for advanced non-small cell lung cancer (NSCLC) patients accepted platinum-based adjuvant treatment was worse than that of GA or AA genotype 31. Similar to those study, our results also showed that chemotherapy GC patients carrying FAS 1377 GA + AA or 670 GA + GG genotypes had poor survival compared with those wide type 39,40. Stratified analyses based on multiple clinical and pathological characteristics were conducted to further identify the potential chemoresistance factors. Li et al. demonstrated that the advanced NSCLC patients with tumor size larger than 3cm had poor response to platinum-based chemotherapy for FAS 670 A > G mutation 31. For GC patients, our results showed that many subgroups, including age, tumor size and site, tumor differentiation, histological types, lymph node metastasis, invasion depth and TNM stage had significant effects on the prognosis of chemotherapy patients with FAS 1377 GA + AA or 670 GA + GG genotypes (**Table 3**). In the combination analysis, a greater than additive effect was identified when patients simultaneously carrying FAS 1377 GA and FAS 670 GA genotypes (HR = 1.830, 95% CI = 1.179-2.840, log-rank *P* < 0.001). This may due to that both the two polymorphisms affected the gene expression level in the same causal pathway, thereby enhancing the synergistic effects 41,42.

Activation-induced cell death (AICD) referred to the apoptosis of tumor-infiltrating lymphocytes (TILs), which may lead to the escape of transformed cells to fight the killing of anti-cancer T lymphocytes, thereby promoting tumor progression 43,44. The AICD of T lymphocytes was a FASL-dependent process and accumulated evidences have demonstrated that the elevated expression of FASL could counterattack FAS-sensitive TILs, which may lead to immune privilege and then weaken the cancer cells' sensitivity to chemotherapy 45. But, recent researches on the relevance between FASL 844 C > T variant with the sensitivity to cancer or the chemotherapy response were controversial. For example, some studies have demonstrated that FASL 844 C > T mutation can diminish cancer development and progression 46,47. Whereas, Li et al. showed that FASL 844 C > T genetic variant had no significant effects on chemotherapy response or survival prognosis for advanced NSCLC patients 31. Similarly, no associations were identified for GC patients accepted platinum-based adjuvant chemotherapy in our investigation (HR = 0.851, 95% CI = 0.561-1.290), but the protection effect may be shown if more patents were enrolled. The explanations for those phenomena were that FASL 844 C > T mutation strongly decreased FASL expression and the AICD process of tumor-specific T cell, which may lead to stronger immune surveillance and then enhance the sensitivity to chemotherapy 48,49. Interestingly, although FAS 1377 GA + GG genotypes could shorten the overall survival time of chemotherapy patients, the poor survival was neutralized when patients simultaneous with FASL 844 TC + TT and FAS 1377 GA + GG genotypes. The similar neutralization was also existed when patients simultaneously carrying FASL 844 TC and FAS 670 GA genotypes (Table 4). The explanations for the above antagonistic effects may due to that the interactions between FASL and FAS could neutralize the effects of the single gene 50,51.

In addition, CASP8 was also a central regulator in FAS-FASL mediated AICD of TILs, but the current researches on the associations between 652 6N I > D variation with patient's survival were debatable 52. For no chemotherapy cohort, Frank et al. has showed that 652 6N D was a risk factor for cancer patients 53. However, many other researchers have revealed that 652 6N del could decrease the cancer risks 54,55. For ovarian cancer patients received chemotherapy, Zhang et al. indicated that the CASP8 652 6N I > D mutation could weaken cisplatin-induced apoptosis 56. However, Li et al. demonstrated 652 6N ins > del variant had a better prognosis for the cutaneous melanoma patients accepted chemotherapy 57. In our investigation, no remarkable association was identified between 652 6N I > D mutation with chemotherapy patient's survival. But the protection effect may be shown if a larger number of patients were enrolled in our study. This might due to 652 6N I > D variation could decrease CASP8 expression and enzyme activity, which could reduce T lymphocytes apoptotic and then increase immune surveillance 57,58. In the combination analysis, the poor survival of patients carrying FAS 670 GA + GG genotype was maintained when CASP8 652 6 II genotypes coexisted at the same time. Interestingly, the inferior survival was neutralized when patients simultaneously carrying CASP8 652 6 DI + DD and FAS 670 GA + GG genotypes, which were similar to the phenomenon for the patients simultaneous with FAS 1377GA and CASP8 652 6DI genotypes. Those neutralization may be caused by the interactions between genes involved in the Fas-induced apoptosis pathway 59,60.

In conclusion, our findings demonstrated that FAS 670 A > G and 1377 G > A mutations lead to a poor reaction to platinum-based adjuvant chemotherapy, which may serve as potential biomarkers to predict the chemotherapy efficacy for stage II-III GC patients. Besides, the gene-gene interactions involved in Fas-mediated apoptosis may enhance or neutralize the chemosensitivity. All in all, our findings provided a strong basis for future prospective clinical trials to choose more effective chemotherapy drugs and design effective personalized therapy regimens for GC patients.

## Supplementary Material

Supplementary figures and tables.

## Figures and Tables

**Table 1:** Clinical characteristics of the gastric cancer patients in two cohorts

Variable	Chemotherapy (261)				No chemotherapy (401)			
	Patients/deaths	MST (mouths)	*P*	HR (95%CI)^a^	Patients/deaths	MST (mouths)	*P*	HR (95%CI)^a^
**Age (years)**								
≤ 60	149/65	62^b^	0.335	1	162/88	48	0.606	1
> 60	112/53	50		1.194 (0.830-1.717)	238/128	50		1.074 (0.817-1.412)
**Sex**								
Male	212/96	62	0.977	1	305/161	53	0.440	1
Female	49/22	55		1.007 (0.633-1.601)	96/56	38		1.126 (0.831-1.527)
**Tumor size**								
≤ 5	143/61	63^b^	0.269	1	212/109	59	0.130	1
> 5	118/57	51		1.224 (0.857-1.756)	189/108	43		1.226 (0.939-1.601)
**Tumor site**								
No-cardia	163/76	50	0.433	1	256/142	41	0.430	1
Cardia	98/42	62^b^		0.861 (0.591-1.255)	145/75	59		0.894 (0.676-1.183)
**Histological type**								
Intestinal	75/37	62	0.333	1	123/50	74	0.006	1
Diffuse	186/81	55		1.100 (0.905-1.336)	278/167	37		1.545 (1.125-2.122)
**Tumor differentiation**								
Well	96/46	62	0.512	1	133/56	70	0.015	1
Poorly	145/63	61^b^		0.804 (0.549-1.176)	231/134	41		1.369 (1.000-1.875)
Mucinous or signet-ring cell	16/7	52		0.813 (0.367-1.802)	21/15	32		2.165 (1.224-3.829)
**Depth of invasion**								
T1/T2	25/15	29	0.108	1	43/17	75^b^	0.045	1
T3/T4	236/103	61^b^		0.645 (0.375-1.110)	353/196	48		1.647 (1.002-2.706)
**Lymph node metastasis**								
NO	54/19	69^b^	0.057	1	89/38	67	0.03	1
N1/N2/N3	207/99	51		1.599 (0.978-2.614)	309/177	41		1.465 (1.032-2.080)
**TNM stage**								
II	75/32	62	0.430	1	127/49	75^b^	<0.001	1
III	186/86	55		1.176 (0.783-1.765)	274/168	37		1.917 (1.394-2.637)

a: Adjusted for age and sex;b: Mean survival time was provided when MST could not be calculated;c: Information was not available for two patients; HR, hazard ratio; CI, confidence interval; MST, median survival time.

**Table 2:** Associations of *FAS*, *FASL* and *Casp8* polymorphisms with gastric cancer patients' survival in both cohorts

Genetic model	Genotypes	Chemotherapy				No chemotherapy			
		Patients/deaths	MST (mouths)	*P*	HR (95%CI)^a^	Patients/Deaths	MST (mouths)	*P*	HR (95%CI)^a^
***FAS rs2234767 1377 G > A***									
Codominant model	GG	113/40	69^b^	0.007	1	147/78	48	0.973	1
	GA	115/60	50		1.734 (1.161-2.589)	198/108	50		1.020 (0.762-1.365)
	AA	26/15	29		2.135 (1.176-3.875)	38/19	88		0.966 (0.585-1.596)
Dominant model	GA/AA	141/75	51	0.002	1.801 (1.225-2.648)	236/127	50	0.936	1.012 (0.763-1.341)
Recessive model	GG/GA	228/100	61^b^	0.091	1	345/186	50	0.849	1
	AA	26/15	29		1.586 (0.920-2.734)	38/19	88		0.955 (0.596-1.532)
***FAS rs1800682 670 A > G***									
Codominant model	AA	94/34	68^b^	0.036	1	120/66	48	0.460	1
	GA	121/62	43		1.702 (1.119-2.590)	214/117	47		1.008 (0.745-1.364)
	GG	36/18	37		1.591 (0.898-2.821)	41/18	62		0.742 (0.440-1.249)
Dominant model	GA/GG	157/80	43	0.010	1.676 (1.120-2.507)	255/135	53	0.794	0.962 (0.716-1.291)
Recessive model	AA/GA	215/96	62	0.547	1	334183	48	0.213	1
	GG	36/18	37		1.166 (0.704-1.929)	41/18	52		0.738 (0.454-1.198)
***FASL rs763110 844 C > T***									
Codominant model	CC	70/31	51^b^	0.536	1	90/39	77	0.418	1
	TC	144/70	52		1.011 (0.656-1.559)	234/134	48		1.248 (0.873-1.784)
	TT	31/13	54^b^		0.471 (0.116-1.918)	43/23	43		1.065 (0.635-1.783)
Dominant model	TC/TT	175/83	55	0.442	0.851 (0.561-1.290)	277/157	47	0.269	0.987 (0.732-1.333)
Recessive model	CC/TC	214/101	52	0.333	1	324/173	53	0.635	1
	TT	31/13	54^b^		0.754 (0.432-1.344)	43/23	43		0.901 (0.583-1.392)
***CASP8 -652 6N ins > del***									
Codominant model	II	153/71	52	0.492	1	226/132	36	0.008	1
	DI	82/38	52^b^		1.037 (0.699-1.538)	143/70	63		0.741 (0.554-0.991)
	DD	13/4	72^b^		0.564 (0.206-1.543)	12/2	100^b^		0.202 (0.054-0.815)
Dominant model	DI/DD	95/42	61^b^	0.832	0.960 (0.655-1.406)	155/72	65	0.011	0.690 (0.518-0.921)
Recessive model	II/DI	235/109	55	0.239	1	369/202	48	0.021	1
	DD	13/4	72^b^		0.557 (0.205-1.510)	12/2	100^b^		0.227 (0.056-0.913)

a: Adjusted for age and sex;b: Mean survival time was provided when MST could not be calculated; I, a six-nucleotide insertion; D; a six-nucleotide deletion.

**Table 3:** Stratified analysis of the FAS 1377 G > A and 670 A > G polymorphisms with gastric cancers' overall survival for the patients received platinum-based chemotherapy

Variables	*FAS 1377 G > A* Genotypes		HR (95% CI)^a^	*P*	*FAS 670 A > G* Genotypes		HR (95% CI)^a^	*P*
	GG	GA/AA			AA	GA/GG		
Total	113/40	141/75	1.801 (1.225-2.648)	0.002	94/34	157/80	1.676 (1.120-2.507)	0.010
**Age**								
≤ 60	67/22	79/64	1.981 (1.181-3.323)	0.008	57/19	85/43	1.852 (1.076-3.188)	0.032
> 60	46/18	62/33	1.558 (0.876-2.773)	0.124	37/15	72/37	1.441 (0.790-2.628)	0.266
**Tumor size**								
≤ 5 cm	69/25	71/35	1.570 (0.938-2.628)	0.081	59/21	80/39	1.614 (0.946-2.752)	0.074
>5 cm	44/15	70/40	2.056 (1.134-3.726)	0.014	35/13	77/41	1.672 (0.895-3.123)	0.099
**Tumor site**								
Non-cardia	66/22	92/52	2.011 (1.220-3.314)	0.005	56/19	100/53	1.806 (1.067-3.055)	0.024
Cardia	47/18	49/23	1.477 (0.795-2.745)	0.211	38/15	57/27	1.469 (0.779-2.770)	0.229
**Histological type**								
Intestinal type	38/14	36/22	1.944 (0.993-3.805)	0.047	31/10	43/27	2.475 (1.192-5.140)	0.011
Diffuse type	75/26	105/53	1.791 (1.118-2.869)	0.013	63/24	114/53	1.406 (0.867-2.280)	0.162
**Tumor differentiation**								
Well to moderately	46/17	49/28	1.761 (0.962-3.223)	0.061	38/13	57/33	2.087 (1.092-3.989)	0.022
Poor	61/21	81/41	1.805 (1.064-3.060)	0.025	51/19	88/41	1.407 (0.816-2.425)	0.214
Mucinous/signet-ring cell	5/1	11/6	3.682 (0.441-30.715)	0.197	4/1	12/6	2.965 (0.352-24.94)	0.295
**Depth of invasion**								
T1/T2	9/5	16/10	1.182 (0.403-3.470)	0.758	7/4	18/11	1.076 (0.342-3.388)	0.899
T3/T4	104/35	125/65	1.851 (1.225-2.795)	0.003	87/30	139/69	1.715 (1.115-2.637)	0.012
**Lymph node metastasis**								
N0	22/4	32/15	3.020 (1.001-9.110)	0.038	19/4	33/15	2.735 (0.902-2.372)	0.063
N1/N2/N3	91/36	109/60	1.678 (1.108-2.541)	0.013	75/30	124/65	1.537 (1.00-2.372)	0.048
**Distant metastasis**								
M0	113/40	141/75	1.801 (1.225-2.646)	0.002	94/34	157/80	1.676 (1.12-2.507)	0.010
**TNM stage**								
II	31/9	44/23	1.073 (0.517-2.226)	0.051	26/8	47/24	2.056 (0.919-4.60)	0.072
III	82/31	97/52	1.707 (1.092-2.670)	0.017	68/26	110/56	1.564 (0.981-2.49)	0.056

a: Adjusted for age and sex;b: Heterogeneity test for differences between groups;c: Information was not available for two patients.

**Table 4:** The effects of the gene-gene interactions on the survival of gastric cancer patients received platinum-based chemotherapy

Combined genotypes	Patients	Deaths	MST (months)	*P*	HR (95%CI)^a^
***FAS 1377 G > A and FAS 670 A > G***					
*FAS 1377 GG + FAS 670 AA*	93	33	69^b^	<0.001	1
*FAS 1377 GG + FAS 670 GA*	19	7	50^b^		1.128 (0.499-2.552)
*FAS 1377GA + FAS 670 AA*	1	1	5		40.84 (4.925-338.6)
*FAS 1377GA + FAS 670 GA*	96	51	43		1.830 (1.179-2.840)
***AS 1377 G > A and FASL 844 C > T***					
*FAS 1377 GG + FASL 844CC*	30	11	57^b^	0.012	1
*FAS 1377 GG + FASL 844TC+TT*	79	28	70		0.734 (0.364-1.480)
*FAS 1377 GA+AA + FASL 844CC*	40	20	23		1.568 (0.751-3.274)
*FAS 1377 GA+AA + FASL 844 TC+TT*	94	53	36		1.449 (0.765-2.777)
***FAS 1377 G > A and CASP8 652 6 ins > del***					
*FAS 1377 GG + CASP8 652 6 II*	73	26	68^b^	0.037	1
*FAS 1377 GG + CASP8 652 6 DI*	32	12	56^b^		1.036 (0.523-2.053)
*FAS 1377 GA + CASP8 652 6 II*	66	38	43		1.918 (1.164-3.162)
*FAS 1377 GA + CASP8 652 6 DI*	39	19	33		1.627 (0.899-2.945)
***FAS 670 A > G* and *FASL 844 C > T***					
*FAS 670AA + FASL 844CC*	23	9	55^b^	0.185	1
*FAS 670AA + FASL 844TC*	56	21	68^b^		0.778 (0.355-1.705)
*FAS 670GA + FASL 844CC*	34	14	32^b^		1.156 (0.500-2.673)
*FAS 670GA+ FASL 844TC*	69	39	35		1.384 (0.670-2.860)
***FAS 670 A > G and CASP8 652 6 ins > del***					
*FAS 670AA + CASP8 652 6 II*	65	23	68^b^	0.059	1
*FAS 670AA + CASP8 652 6 DD+DI*	27	10	70^b^		0.984 (0.468-2.068)
*FAS 670 GA+GG + CASP8 652 6 II*	87	47	43		1.790 (1.086-2.950)
*FAS 670 GA+GG + CASP8 652 6 DD+DI*	65	31	37		1.630 (0.948-2.801)

a: Adjusted for age and sex;b: Mean survival time was provided when MST could not be calculated.

## Abbreviations

GC: Gastric Cancer
SNPs: single nucleotide polymorphisms
TNF: tumor necrosis factor
Sp1: Transcription factor Sp1
STAT1: Signal Transducer and Activator of Transcription 1
CTL: Cytotoxic T Lymphocytes
MST: Median Survival Time
HRs: Hazard Ratios
95%CIs: 95% Confidence Intervals
AICD: Activation-Induced Cell Death
TILs: Tumor-Infiltrating Lymphocytes
