# Supplementary material for: Genomic variants in Fas-mediated apoptosis pathway predict a poor response to Platinum-based Chemotherapy for Chinese Gastric Cancer Patients
Source: J Cancer. 2021 Jan 1;12(3):849–59. doi: 10.7150/jca.48120 (PMC7778532; doi:10.7150/jca.48120)
Supplement: Supplementary file 1 — Supplementary figures and tables. [file jcav12p0849s1.pdf]

Supplementary Table S1. The effects of gene-gene interactions in homozygous model on the survival of gastric cancer patients receiving platinum-based chemotherapy

combined genotypes	patient	death	MST	P	HR (95%CI)[a]
FAS 1377 G> A **and** ***FAS 670 A> G***					
FAS 1377 GG + FAS 670 AA	208	96		0.790	1
FAS 1377 GG + FAS 670 GG	2	1			1.398(0.195-10.036)
FAS 1377AA + FAS 670 AA	1	0			0
FAS 1377AA + FAS 670 GG	51	24			1.112(0.711-1.741)
FAS 1377 G> A **and** ***FASL 844 C> T***					
FAS 1377 GG + FASL 844CC	30	11		0.074	1
FAS 1377 GG + FASL 844TT	16	6			0.691(0.252-1.900)
FAS 1377AA + FASL 844CC	9	6			2.508(0.922-6.823)
FAS 1377AA + FASL 844TT	2	0			0
FAS 1377 G> A **and** ***CASP8 6526 ins > del***					
FAS 1377 GG + CASP8 6526 II	73	26	68[b]	0.434	1
FAS 1377 GG + CASP8 6526 DD	6	1	87[b]		0.378(0.051-2.788)
FAS 1377AA + CASP8 6526 II	12	6	30		1.691(0.693-4.127)
FAS 1377AA + CASP8 6526 DD	2	1	18		1.470(0.199-10.863)
FAS 670 A> G **and** ***FASL 844 C> T***					
FAS 670AA + FASL 844CC	23	9	55[b]	0.218	1
FAS 670AA + FASL 844TT	13	4	62[b]		0.526(0.160-1.730)
FAS 670GG + FASL 844CC	13	8	19		1.737(0.668-4.520)
FAS 670GG + FASL 844TT	3	1	36		0.688(0.087-5.456)
FAS 670 A> G **and** ***CASP8 6526 ins > del***					
FAS 670AA + CASP8 6526 II	65	23	68[b]	0.622	1
FAS 670AA + CASP8 6526 DD	5	1	87[b]		0.430(0.058-3.192)
FAS 670GG + CASP8 6526 II	19	9	55		1.384(0.640-2.993)
FAS 670GG + CASP8 6526 DD	2	1	18		1.553(0.209-11.549)
FASL 844 C> T **and** ***CASP8 6526 ins > del***					
FASL 844CC + CASP8 6526 II	40	19		0.477	1

FASL 844CC + CASP8 6526 DD	4	2			1.025(0.238-4.410)
FASL 844TT + CASP8 6526 II	19	8			0.635(0.276-1.460)
FASL 844TT + CASP8 6526 DD	2	0			0

[a]Adjusted for age and sex.

[b]Mean survival time was provided when MST could not be calculated.

Supplementary table S2. The effects of gene-gene interactions in heterozygous model on the survival of gastric cancer patients receiving platinum-based chemotherapy

combined genotypes	patient	death	MST	P	HR (95%CI)[a]
FAS 1377 G> A** and **FAS 670 A> G					
FAS 1377 GG + FAS 670 AA	93	33	69[b]	<0.001	1
FAS 1377 GG + FAS 670 GA	19	7	50[b]		1.128(0.499-2.552)
FAS 1377GA + FAS 670 AA	1	1	5		40.84(4.925-338.6)
FAS 1377GA + FAS 670 GA	96	51	43		1.830(1.179-2.840)
AS 1377 G> A** and **FASL 844 C> T					
FAS 1377 GG + FASL 844CC	30	11	57[b]	0.049	1
FAS 1377 GG + FASL 844TC	63	22	70[b]		0.762(0.368-1.579)
FAS 1377 GA + FASL 844CC	31	14	36[b]		1.357(0.616-2.990)
FAS 1377 GA + FASL 844TC	68	40	35		1.533(0.786-2.993)
FAS 1377 G> A** and **CASP8 6526 ins > del					
FAS 1377 GG + CASP8 6526 II	73	26	68[b]	0.037	1
FAS 1377 GG + CASP8 6526 DI	32	12	56[b]		1.036(0.523-2.053)
FAS 1377 GA + CASP8 6526 II	66	38	43		1.918(1.164-3.162)
FAS 1377 GA + CASP8 6526 DI	39	19	33		1.627(0.899-2.945)
FAS 670 A> G and ***FASL 844 C> T***					
FAS 670AA + FASL 844CC	23	9	55[b]	0.185	1
FAS 670AA + FASL 844TC	56	21	68[b]		0.778(0.355-1.705)
FAS 670GA + FASL 844CC	34	14	32[b]		1.156(0.500-2.673)
FAS 670GA+ FASL 844TC	69	39	35		1.384(0.670-2.860)
FAS 670 A> G** and **CASP8 6526 ins > del					
FAS 670AA + CASP8 6526 II	65	23	68[b]	0.075	1
FAS 670AA + CASP8 6526 DI	22	9	54[b]		1.148(0.531-2.481)
FAS 670GA+ CASP8 6526 II	68	38	43		1.904(1.133-3.198)
FAS 670GA + CASP8 6526 DI	42	20	51[b]		1.593(0.873-2.907)
FASL 844 C> T** and **CASP8 6526 ins > del					
FASL 844CC + CASP8 6526 II	40	19	49[b]	0.803	1

FASL 844CC + CASP8 6526 DI	25	9	33[b]		0.733(0.331-1.621)
FASL 844TC + CASP8 6526 II	89	43	52		0.842(0.490-1.448)
FASL 844TC + CASP8 6526 DI	44	24	30		0.984(0.538-1.799)

[a]Adjusted for age and sex.

[b]Mean survival time was provided when MST could not be calculated.

Supplementary Table S3. The effects of gene-gene interactions in dominant model on the survival of gastric cancer patients receiving platinum-based chemotherapy

combined genotypes	patient	death	MST	*P*	HR (95%CI)[a]
FAS 1377 G> A** and **FAS 670 A> G					
FAS 1377 GG + FAS 670 AA	93	33	69[b]	<0.001	1
FAS 1377 GG + FAS 670 GA+GG	19	7	50[b]		1.132(0.501-2.559)
FAS 1377 GA+AA + FAS 670 AA	1	1	5		39.245(4.86-316.6)
FAS 1377 GA+AA + FAS 670 GA+GG	135	71	43		1.808(1.195-2.737)
AS 1377 G> A** and **FASL 844 C> T					
FAS 1377 GG + FASL 844CC	30	11	57[b]	0.012	1
FAS 1377 GG + FASL 844TC+TT	79	28	70		0.734(0.364-1.480)
FAS 1377 GA+AA + FASL 844CC	40	20	23		1.568(0.751-3.274)
FAS 1377 GA+AA + FASL 844 TC+TT	94	53	36		1.449(0.765-2.777)
FAS 1377 G> A** and **CASP8 6526 ins > del					
FAS 1377 GG + CASP8 6526 II	73	26	68[b]	0.012	1
FAS 1377 GG + CASP8 6526 DD+DI	38	13	71[b]		0.912(0.469-1.775)
FAS 1377 GA+AA + CASP8 6526 II	78	44	43		1.889(1.162-3.070)
FAS 1377 GA+AA + CASP8 6526 DD+DI	57	29	33		1.757(1.033-2.990)
FAS 670 A> G and ***FASL 844 C> T***					
FAS 670AA + FASL 844CC	23	9	55[b]	0.068	1
FAS 670 AA + FASL 844TC+TT	69	25	70[b]		0.704(0.328-1.513)
FAS 670 GA+GG + FASL 844CC	47	22	35[b]		1.322(0.608-2.873)
FAS 670 GA+GG + FASL 844 TC+TT	105	57	39		1.263(0.625-2.554)
FAS 670 A> G** and **CASP8 6526 ins > del					
FAS 670AA + CASP8 6526 II	65	23	68[b]	0.059	1
FAS 670AA + CASP8 6526 DD+DI	27	10	70[b]		0.984(0.468-2.068)
FAS 670 GA+GG + CASP8 6526 II	87	47	43		1.790(1.086-2.950)
FAS 670 GA+GG + CASP8 6526 DD+DI	65	31	37		1.630(0.948-2.801)
FASL 844 C> T** and **CASP8 6526 ins > del					
FASL 844CC + CASP8 6526 II	40	19	49[b]	0.833	1

FASL 844CC + CASP8 6526 DD+DI	29	11	32[b]		0.782(0.372-1.644)
FASL 844 TC+TT + CASP8 6526 II	108	51	52		0.785(0.463-1.333)
FASL 844 TC+TT + CASP8 6526 DD+DI	61	30	51		0.824(0.463-1.466)

[a]Adjusted for age and sex.

[b]Mean survival time was provided when MST could not be calculated.

Supplementary Table S4. Primers for the genotyping assays of Fas, FasL, CASP8

Genotypes	Forward primer	Reverse primer	Extension primer
FAS rs2234767 1377 G > A	TTTTGTGTCTATTAGATGCTCAGAGTGTGTG	gactgttagtgccatgaggAAGACC	TTTTTTTTTCCATGAGGAAGACCCTGGG
FAS rs1800682 670 A > G	TTGTGGCTGCAACATGAGAGG	TGGCGCAACATCTGTACTTTTTCA	TTTTTTTTTTTTTTTCATGAGAGGCTCACRGACGTT
FASL rs763110 C > T	taaataaaCTGGGCAAACAATGAAAATG	AAACCAGTGGAACCCACAGAGC	TTTTTTTTTTTTTTTTTTTTTTTTTTTTGCAAACAATGAAAATGAAAACATTG
CASP8 -652 6N ins > del	ccgaggaaggcactgagacg	GCTTAWAAAGCCATAGTaattcttgctctgc	
